# Estimation of different types of entropies for the Kumaraswamy distribution

**DOI:** 10.1371/journal.pone.0249027

**Published:** 2021-03-30

**Authors:** Abdulhakim A. Al-Babtain, Ibrahim Elbatal, Christophe Chesneau, Mohammed Elgarhy

**Affiliations:** 1 Department of Statistics and Operations Research, King Saud University, Riyadh, Saudi Arabia; 2 Department of Mathematics and Statistics, College of Science, Imam Mohammad Ibn Saud Islamic University (IMSIU), Riyadh, Saudi Arabia; 3 Department of Mathematics, Université de Caen, LMNO, Campus II, Science 3, Caen, France; 4 The Higher Institute of Commercial Sciences, Al mahalla Al kubra, Algarbia, Egypt; Tongii University, CHINA

## Abstract

The estimation of the entropy of a random system or process is of interest in many scientific applications. The aim of this article is the analysis of the entropy of the famous Kumaraswamy distribution, an aspect which has not been the subject of particular attention previously as surprising as it may seem. With this in mind, six different entropy measures are considered and expressed analytically via the beta function. A numerical study is performed to discuss the behavior of these measures. Subsequently, we investigate their estimation through a semi-parametric approach combining the obtained expressions and the maximum likelihood estimation approach. Maximum likelihood estimates for the considered entropy measures are thus derived. The convergence properties of these estimates are proved through a simulated data, showing their numerical efficiency. Concrete applications to two real data sets are provided.

## 1 Introduction

Information theory provides natural mathematical tools for measuring the uncertainty of random variables and the information shared by them. In this regard, entropy and mutual information are two fundamental concepts. More precisely, the probability distribution of a random variable is associated with some sort of uncertainty, and entropy is used to quantify it. The concept of entropy was formerly proposed by [1]. Since that publication, many areas of study such as statistics, neurobiology, cryptography, bioinformatics, quantum computer science and linguistics, have developed various entropy-based measures. Modern and exhaustive reviews on the ‘entropy universe’ can be found in [2–6].

In applied probability and statistics, many authors have conducted their studies for diverse and important distributions based on entropy. The essential references in this regard are briefly presented below. Reference [7] used the concept of entropy to communicate on the probability distribution of electric charge between atoms observed in a certain condition. Reference [8] derived the entropy for the Feller-Pareto family and presented the entropy ordering property for some related sample minimum and maximum. Reference [9] estimated the entropy of the Weibull distribution by considering different loss functions based on a generalized progressively hybrid censoring scheme. Reference [10] discussed the entropy for the generalized half-logistic distribution based on the type II censored samples. References [11] and [12] proposed estimates for the entropy of absolutely continuous random variables. Reference [13] presented an indirect method using a decomposition to simplify the entropy’s calculation under the progressive type II censoring. Reference [14] derived a nonparametric kernel estimator for the general Shannon entropy. Reference [15] estimated the entropy for several exponential distributions and extended the results to other circumstances. Reference [16] estimated the Shannon entropy of the Rayleigh model under doubly generalized type-II hybrid censoring, and evaluated its performance by two criteria. Reference [17] derived a nonparametric wavelet estimator for the general Shannon entropy. Reference [18] provided an exact expression for entropy information contained in both types of progressively hybrid censored data and applied it in the setting of the exponential distribution. Reference [19] investigated entropy measures for weighted and truncated weighted exponential distributions. Reference [20] presented the estimation of entropy for inverse Weibull distribution under multiple censored data. Reference [21] introduced estimation of entropy for inverse Lomax distribution under the multiple censored scheme. Reference [22] examined Bayesian and non-Bayesian methods to estimate the dynamic cumulative residual Rényi entropy for the Lomax distribution.

Surprisingly, to our knowledge, the entropy of the famous Kumaraswamy distribution has not been studied in depth. In this article, we fill this gap both probabilistically and statistically. The specificities and interests of the Kumaraswamy distribution are described below. First, it was introduced by [23], and was motivated as an alternative to the beta distribution which are (i) mathematically simpler, without special function in particular, and (ii) more suited to the modeling of various hydrological phenomena observed at low frequency (daily rainfall, daily flow of rivers, etc.). Mathematically, the probability density function (pdf) of the Kumaraswamy distribution is specified by

$$\begin{array}{c} f\left(x;a,b\right)=abx^{a-1}{\left(1-x^{a}\right)}^{b-1},\;0<x<1, \end{array}$$ (1)

with *f*(*x*;*a*, *b*) = 0 otherwise, where *a*, *b* > 0. This pdf is unimodal if *a*, *b* > 1, uniantimodal if *a*, *b* < 1, increasing if *a* > 1, *b* ≤ 1, decreasing if *a* ≤ 1, *b* > 1 or constant if *a* = *b* = 1, in the same way as the beta distribution. The corresponding cumulative distribution and quantile functions are quite simple; they are defined without special function contrary to those of the beta distribution. Special cases of the Kumaraswamy distribution correspond to the distribution of minimum or maximum of uniform samples. We may refer the reader to [24] for all the known features of this distribution. Also, the kumaraswamy distribution has generated many flexible distributions with various domains and number of parameters through the generalized Kumaraswamy class elaborated by [25].

In a sense, this study complements the work of [24] by investigating the overall concept of entropy of the Kumaraswamy distribution, which has never been studied before. More precisely, we consider six well-referenced entropy measures. We derive their analytical expressions by using the well-known beta function. We compare them numerically by considering different parameter values. Then, we propose an efficient strategy based on the maximum likelihood approach to estimate these entropy measures. A simulation study is done to see how effective our strategy is. Graphical and numerical comparisons are performed. We end the study by two illustrative examples on real data sets, showing how the methodology can be applied in a concrete statistical setting.

The following sections make up the document. Section 2 presents a result on a special integral, and shows how it is related to important entropy measures of the Kumaraswamy distribution. Numerical values of these entropy measures with different values of the parameters are also given. Section 3 studies the estimation of these entropy measures. Then, using generated values from the Kumaraswamy distribution, graphical and numerical comparisons are discussed. The entropy of the random characteristics behind two real data sets is investigated. Finally, conclusions are presented in section 4.

## 2 Entropy of the Kumaraswamy distribution

### 2.1 An integral result

The following result shows that a certain integral involving the pdf of the Kumaraswamy distribution can be expressed in terms of the classical beta function. The connection between this integral and the considered entropy measures will be developed later.

**Proposition 1** *Let δ* > 0, *f*(*x*;*a*, *b*) *be specified by* Eq (1) *and*

$$\begin{array}{c} I_{\delta }\left(a,b\right)=\int_{0}^{1}f{\left(x;a,b\right)}^{\delta }dx. \end{array}$$

*Then, I*_*δ*_(*a*, *b*) *exists if and only if* min(*a*, *b*)>max(1 − 1/*δ*, 0), *and it is expressed as*

$$\begin{array}{c} I_{\delta }\left(a,b\right)=b^{\delta }a^{\delta -1}B(\delta (1-\frac{1}{a})+\frac{1}{a},\delta \left(b-1\right)+1), \end{array}$$

*where B*(*u*, *v*) *denotes the classical beta function, that is* $B(u,v)=\int_{0}^{1}x^{u-1}(1-x{)}^{v-1}dx$ *for u*, *v* > 0.

**Proof**. Owing to Eq (1), we have

$$\begin{array}{c} I_{\delta }\left(a,b\right)=\int_{0}^{1}f{\left(x;a,b\right)}^{\delta }dx={\left(ab\right)}^{\delta }\int_{0}^{1}x^{\delta (a-1)}{\left(1-x^{a}\right)}^{\delta (b-1)}dx. \end{array}$$

When *x* tends to 0, we have *x*^*δ*(*a*−1)^(1 − *x*^*a*^)^*δ*(*b*−1)^ ∼ *x*^*δ*(*a*−1)^, which is integrable in the neighborhood of 0 if and only if *δ*(1 − *a*)<1 by the Riemann integral criteria. Similarly, when *x* tends to 1, we have

$$\begin{array}{c} x^{\delta (a-1)}{\left(1-x^{a}\right)}^{\delta (b-1)}∼{\left(1-x^{a}\right)}^{\delta (b-1)}∼a^{\delta (b-1)}{\left(1-x\right)}^{\delta (b-1)}, \end{array}$$

which is integrable in the neighborhood of 1 if and only if *δ*(1 − *b*)<1 by the Riemann integral criteria. In summary, *I*_*δ*_(*a*, *b*) exists if and only if *δ*max(1 − *a*, 1 − *b*)<1, which is equivalent to min(*a*, *b*)>1 − 1/*δ*. Now, under this assumption, by applying the change of variables *y* = *x*^*a*^, that is $x=y^{\frac{1}{a}}$ with $dx=y^{\frac{1}{a}-1}dy/a$, we obtain

$$\begin{array}{cc} I_{\delta }\left(a,b\right) & ={\left(ab\right)}^{\delta }\int_{0}^{1}x^{\delta (a-1)}{\left(1-x^{a}\right)}^{\delta (b-1)}dx=b^{\delta }a^{\delta -1}\int_{0}^{1}y^{\delta (1-\frac{1}{a})+\frac{1}{a}-1}{\left(1-y\right)}^{\delta (b-1)}dy\\ & =b^{\delta }a^{\delta -1}B(\delta (1-\frac{1}{a})+\frac{1}{a},\delta \left(b-1\right)+1). \end{array}$$

This ends the proof of Proposition 1.

In fact, the beta function is implemented in most of the mathematical software (see the function beta of the package stat of R, the Beta function of Mathematica, etc.). Therefore, thanks to Proposition 1, the computation of *I*_*δ*_(*a*, *b*) can be done quite efficiently with little effort. Also, the existing results on the beta functions allow a mathematical control of this integral. Some related results are presented below.

- Through the use of the standard Euler gamma function given as $\Gamma (u)=\int_{0}^{+\infty }x^{u-1}e^{-x}dx$, one can write
  $$\begin{array}{c} I_{\delta }\left(a,b\right)=b^{\delta }a^{\delta -1}\frac{\Gamma (\delta (1-1/a)+1/a)\Gamma (\delta (b-1)+1)}{\Gamma (\delta (b-1/a)+1/a+1)}. \end{array}$$
- Also, assuming that *δ*(1 − 1/*a*) + 1/*a* and *δ*(*b* − 1) + 1 are positive integers, the following formula holds:
  $$\begin{array}{cc} I_{\delta }\left(a,b\right) & =b^{\delta }a^{\delta -1}\frac{[\delta (1-1/a)+1/a-1]![\delta (b-1)]!}{[\delta (b-1/a)+1/a]!}. \end{array}$$
- By virtue of the main result in [26], if *δ*(*a* − 1) ≥ *a* − 1 and *b* ≥ 1, then we have
  $$\begin{array}{c} \alpha b^{\delta }a^{\delta -1}\leq \frac{b^{\delta }a^{\delta }}{\left[\delta (a-1)+1][\delta (b-1)+1\right]}-I_{\delta }\left(a,b\right)\leq \beta b^{\delta }a^{\delta -1}, \end{array}$$
  with the best possible constants *α* = 0 and *β* = 0.08731…. Therefore, for not too large value of *δ*, the following numerical approximation seems acceptable:
  $$\begin{array}{c} I_{\delta }\left(a,b\right)\approx \frac{b^{\delta }a^{\delta }}{\left[\delta (a-1)+1][\delta (b-1)+1\right]}. \end{array}$$

In our study, the interest of Proposition 1 is that *I*_*δ*_(*a*, *b*) is the main ingredient in the definitions of various entropy measures of the Kumaraswamy distribution, as developed in the next part.

### 2.2 Various entropy measures

The entropy of the Kumaraswamy distribution can be measured in different manners. The most useful entropy measures of the literature are recalled in Table 1 for a general distribution with pdf denoted by *f*(*x*;*φ*), *φ* representing a possible vector of parameters. Also, we suppose that *δ* > 0 and *δ* ≠ 1 as basic assumptions in this general case.

**Table 1:** Important entropy measures of a distribution with pdf *f*(*x*;*φ*) at *δ*.

Name of the entropy	Reference	Notation	Expression
Rényi	[28]	*R*_*δ*_(*φ*)	$\frac{1}{1-\delta }\text{log}\left[\int_{-\infty }^{+\infty }f(x;\varphi {)}^{\delta }dx\right]$
Havrda and Charvat	[29]	*HC*_*δ*_(*φ*)	$\frac{1}{2^{1-\delta }-1}\left[\int_{-\infty }^{+\infty }f(x;\varphi {)}^{\delta }dx-1\right]$
Arimoto	[30]	*A*_*δ*_(*φ*)	$\frac{\delta }{1-\delta }\left\{{\left[\int_{-\infty }^{+\infty }f(x;\varphi {)}^{\delta }dx\right]}^{\frac{1}{\delta }}-1\right\}$
Tsallis	[31]	*T*_*δ*_(*φ*)	$\frac{1}{\delta -1}\left[1-\int_{-\infty }^{+\infty }f(x;\varphi {)}^{\delta }dx\right]$
Awad and Alawneh 1	[27]	*AA*1_*δ*_(*φ*)	$\frac{1}{\delta -1}\text{log}\left\{{\left[\underset{x\in \mathbb{R}}{\text{sup}}f(x;\varphi )\right]}^{1-\delta }\int_{-\infty }^{+\infty }f(x;\varphi {)}^{\delta }dx\right\}$
Awad and Alawneh 2	[27]	*AA*2_*δ*_(*φ*)	$\frac{1}{2^{1-\delta }-1}\left[\left\{{\left[\underset{x\in \mathbb{R}}{\text{sup}}f(x;\varphi )\right]}^{1-\delta }\int_{-\infty }^{+\infty }f(x;\varphi {)}^{\delta }dx\right\}-1\right]$

For the two entropy measures proposed by [27], it is supposed that ${\text{sup}}_{x\in \mathbb{R}}f(x;\varphi )$ is finite and well identified.

From Table 1, we see that the integral $\int_{-\infty }^{+\infty }f(x;\varphi {)}^{\delta }dx$ is central to determine the considered entropy measures. Now, we present the corresponding entropy measures of the Kumaraswamy distribution. Based on Proposition 1, it is supposed that *a*, *b* and *δ* satisfy min(*a*, *b*)>max(1 − 1/*δ*, 0).

#### Rényi entropy

Based on Table 1, Eq (1) and Proposition 1, the Rényi entropy of the Kumaraswamy distribution can be expressed as

$$\begin{array}{cc} R_{\delta }\left(a,b\right) & =\frac{1}{1-\delta }\log[I_{\delta }\left(a,b\right)]\\ & =\frac{1}{1-\delta }\{\delta \log b+\left(\delta -1\right)\log a+\log[B(\delta (1-\frac{1}{a})+\frac{1}{a},\delta \left(b-1\right)+1)]\}. \end{array}$$

#### Havrda and Charvát entropy

From Table 1, Eq (1) and Proposition 1, the Havrda and Charvát entropy of the Kumaraswamy distribution can be expressed as

$$\begin{array}{cc} HC_{\delta }\left(a,b\right) & =\frac{1}{2^{1-\delta }-1}[I_{\delta }\left(a,b\right)-1]\\ & =\frac{1}{2^{1-\delta }-1}[b^{\delta }a^{\delta -1}B(\delta (1-\frac{1}{a})+\frac{1}{a},\delta \left(b-1\right)+1)-1]. \end{array}$$

#### Arimoto entropy

Again, from Table 1, Eq (1) and Proposition 1, the Arimoto entropy of the Kumaraswamy distribution is specified by

$$\begin{array}{cc} A_{\delta }\left(a,b\right) & =\frac{\delta }{1-\delta }[I_{\delta }{\left(a,b\right)}^{\frac{1}{\delta }}-1]\\ & =\frac{\delta }{1-\delta }\{ba^{1-\frac{1}{\delta }}{\left[B(\delta (1-\frac{1}{a})+\frac{1}{a},\delta \left(b-1\right)+1)\right]}^{\frac{1}{\delta }}-1\}. \end{array}$$

#### Tsallis entropy

Based on Table 1, Eq (1) and Proposition 1, the Tsallis entropy of the Kumaraswamy distribution can be expressed as

$$\begin{array}{cc} T_{\delta }\left(a,b\right) & =\frac{1}{\delta -1}[1-I_{\delta }\left(a,b\right)]\\ & =\frac{1}{\delta -1}[1-b^{\delta }a^{\delta -1}B(\delta (1-\frac{1}{a})+\frac{1}{a},\delta \left(b-1\right)+1)]. \end{array}$$

#### Awad and Alawneh 1 entropy

From Table 1, Eq (1) and Proposition 1, the Awad and Alawneh 1 entropy of the Kumaraswamy distribution is given as

$$\begin{array}{c} AA1_{\delta }\left(a,b\right)=\frac{1}{\delta -1}\log\{{\left[\underset{0<x<1}{\sup}f\left(x;a,b\right)\right]}^{1-\delta }I_{\delta }\left(a,b\right)\}. \end{array}$$ (2)

Before going further, we need to determine sup_0<*x*<1_ *f*(*x*;*a*, *b*). The following lemma provides the necessary in this regard.

**Lemma 2** *Let f*(*x*;*a*, *b*) *be given as* Eq (1). *Then*, sup_0<*x*<1_ *f*(*x*;*a*, *b*) *is finite if and only if a* ≥ 1 *and b* ≥ 1 *with ab* ≠ 1, *and in this case, we have*

$$\begin{array}{c} \underset{0<x<1}{\sup}f\left(x;a,b\right)=a^{b}b{\left(a-1\right)}^{1-\frac{1}{a}}{\left(b-1\right)}^{b-1}{\left(ab-1\right)}^{\frac{1}{a}-b}. \end{array}$$

**Proof**. We have

$$\begin{array}{cc} f^{\prime }\left(x;a,b\right) & =ab\left(a-1\right)x^{a-2}{\left(1-x^{a}\right)}^{b-1}-a^{2}b\left(b-1\right)x^{2a-2}{\left(1-x^{a}\right)}^{b-2}\\ & =abx^{a-2}{\left(1-x^{a}\right)}^{b-2}\{\left(a-1\right)-[\left(a-1)+a(b-1\right)]x^{a}\}. \end{array}$$

Therefore, *f*′(x_*_;*a*, *b*) = 0 implies that

$$\begin{array}{c} x_{*}={\left(\frac{a-1}{ab-1}\right)}^{\frac{1}{a}}. \end{array}$$

Since *f*′(*x*;*a*, *b*)>0 for *x* < x_*_ and *f*′(*x*;*a*, *b*)<0 for *x* > x_*_, x_*_ is a maximum point for *f*(*x*;*a*, *b*). Hence,

$$\begin{array}{cc} \underset{0<x<1}{\sup}f\left(x;a,b\right) & =f\left(x_{*};a,b\right)=abx_{*}^{a-1}{\left(1-x_{*}^{a}\right)}^{b-1}\\ & =ab{\left(\frac{a-1}{ab-1}\right)}^{1-\frac{1}{a}}{\left(\frac{a(b-1)}{ab-1}\right)}^{b-1}\\ & =a^{b}b{\left(a-1\right)}^{1-\frac{1}{a}}{\left(b-1\right)}^{b-1}{\left(ab-1\right)}^{\frac{1}{a}-b}. \end{array}$$

Note that, for *a* = 1, with the convention 0^0^ = 1, we have *f*(x_*_;*a*, *b*) = *b*(*b* − 1)^*b*−1^(*b* − 1)^1−*b*^ = *b* and for *b* = 1, we have $f(x_{*};a,b)=a(a-1{)}^{1-\frac{1}{a}}(a-1{)}^{\frac{1}{a}-1}=a$. This ends the proof of Lemma 2.

Based on Lemma 2, if *a* > 1 and *b* > 1, Eq (2) becomes

$$\begin{array}{cc} & AA1_{\delta }\left(a,b\right)=\frac{1}{\delta -1}\log\{a^{\left(b-1)(1-\delta \right)}b{\left(a-1\right)}^{\left(1-\delta \right)(1-\frac{1}{a})}{\left(b-1\right)}^{\left(1-\delta )(b-1\right)}\times \\ & {\left(ab-1\right)}^{\left(1-\delta \right)(\frac{1}{a}-b)}B(\delta (1-\frac{1}{a})+\frac{1}{a},\delta \left(b-1\right)+1)\}\\ & =\frac{1}{\delta -1}\{\left(b-1\right)\left(1-\delta \right)\log a+\log b+\left(1-\delta \right)(1-\frac{1}{a})\log\left(a-1\right)\\ & +\left(1-\delta \right)\left(b-1\right)\log\left(b-1\right)+\left(1-\delta \right)(\frac{1}{a}-b)\log\left(ab-1\right)\\ & +\log[B(\delta (1-\frac{1}{a})+\frac{1}{a},\delta \left(b-1\right)+1)]\}. \end{array}$$

#### Awad and Alawneh 2 entropy

From Table 1, Eq (1), Proposition 1 and Lemma 2, the Awad and Alawneh 2 entropy of the Kumaraswamy distribution is given as

$$\begin{array}{cc} AA2_{\delta } & =\frac{1}{2^{1-\delta }-1}[\{a^{\left(b-1)(1-\delta \right)}b{\left(a-1\right)}^{\left(1-\delta \right)(1-\frac{1}{a})}{\left(b-1\right)}^{\left(1-\delta )(b-1\right)}\times \\ & {\left(ab-1\right)}^{\left(1-\delta \right)(\frac{1}{a}-b)}B(\delta (1-\frac{1}{a})+\frac{1}{a},\delta \left(b-1\right)+1)\}-1]. \end{array}$$

Theoretically, it is complicated to study the behavior of these entropy measures. For this reason, a numerical study is proposed in the next section.

### 2.3 Numerical values

We now investigate the numerical values for the six entropy measures presented in Subsection 2.2 under the following configuration of the parameters: Configuration 1: *a* = 2, *b* ∈ *Υ* with *Υ* = {1.5, 2.0, 2.5, 3.0, 3.5, 4.0, 4.5, 5.0, 5.5, 6.0} and *δ* = 0.5, Configuration 2: *a* = 2, *b* ∈ *Υ* and *δ* = 1.5, Configuration 3: *a* = 2, *b* ∈ *Υ* and *δ* = 2.5, Configuration 4: *a* ∈ *Υ*, *b* = 2 and *δ* = 0.5, Configuration 5: *a* ∈ *Υ*, *b* = 2 and *δ* = 1.5, and Configuration 6: *a* ∈ *Υ*, *b* = 2 and *δ* = 2.5. The findings of all the six entropy measures are presented for these configurations in Tables 2–7, respectively.

**Table 2:** Numerical values of the considered entropy measures of the Kumaraswamy distribution at *a* = 2 and *δ* = 0.5.

*b*	*R*_*δ*_(*a*, *b*)	*HC*_*δ*_(*a*, *b*)	*A*_*δ*_(*a*, *b*)	*T*_*δ*_(*a*, *b*)	*AA*1_*δ*_(*a*, *b*)	*AA*2_*δ*_(*a*, *b*)
1.5	-0.034	-0.092	-0.075	-0.076	-0.142	0.430
2.0	-0.037	-0.100	-0.081	-0.083	-0.151	0.457
2.5	-0.047	-0.127	-0.103	-0.106	-0.163	0.500
3.0	-0.060	-0.161	-0.129	-0.134	-0.175	0.538
3.5	-0.074	-0.197	-0.157	-0.163	-0.184	0.570
4.0	-0.088	-0.233	-0.183	-0.193	-0.192	0.596
4.5	-0.102	-0.267	-0.209	-0.221	-0.198	0.618
5.0	-0.115	-0.299	-0.232	-0.248	-0.203	0.637
5.5	-0.128	-0.330	-0.255	-0.273	-0.208	0.653
6.0	-0.140	-0.359	-0.275	-0.297	-0.212	0.667

**Table 3:** Numerical values of the considered entropy measures of the Kumaraswamy distribution at *a* = 2 and *δ* = 1.5.

*b*	*R*_*δ*_(*a*, *b*)	*HC*_*δ*_(*a*, *b*)	*A*_*δ*_(*a*, *b*)	*T*_*δ*_(*a*, *b*)	*AA*1_*δ*_(*a*, *b*)	*AA*2_*δ*_(*a*, *b*)
1.5	-0.069	-0.280	-0.162	-0.164	-0.108	0.398
2.0	-0.075	-0.306	-0.177	-0.179	-0.113	0.416
2.5	-0.091	-0.377	-0.217	-0.221	-0.120	0.439
3.0	-0.110	-0.461	-0.264	-0.270	-0.125	0.457
3.5	-0.129	-0.547	-0.312	-0.320	-0.129	0.471
4.0	-0.147	-0.631	-0.359	-0.370	-0.132	0.483
4.5	-0.165	-0.713	-0.404	-0.418	-0.135	0.491
5.0	-0.181	-0.792	-0.447	-0.464	-0.137	0.499
5.5	-0.197	-0.867	-0.488	-0.508	-0.139	0.505
6.0	-0.211	-0.939	-0.528	-0.550	-0.140	0.510

**Table 4:** Numerical values of the considered entropy measures of the Kumaraswamy distribution at *a* = 2 and *δ* = 2.5.

*b*	*R*_*δ*_(*a*, *b*)	*HC*_*δ*_(*a*, *b*)	*A*_*δ*_(*a*, *b*)	*T*_*δ*_(*a*, *b*)	*AA*1_*δ*_(*a*, *b*)	*AA*2_*δ*_(*a*, *b*)
1.5	-0.087	-0.546	-0.214	-0.235	-0.089	0.408
2.0	-0.095	-0.600	-0.233	-0.259	-0.093	0.423
2.5	-0.114	-0.743	-0.283	-0.320	-0.097	0.440
3.0	-0.134	-0.913	-0.340	-0.393	-0.101	0.454
3.5	-0.155	-1.094	-0.398	-0.471	-0.103	0.464
4.0	-0.174	-1.278	-0.454	-0.551	-0.105	0.472
4.5	-0.193	-1.463	-0.509	-0.631	-0.107	0.478
5.0	-0.210	-1.647	-0.561	-0.710	-0.108	0.483
5.5	-0.226	-1.830	-0.611	-0.789	-0.109	0.487
6.0	-0.241	-2.011	-0.659	-0.867	-0.110	0.490

**Table 5:** Numerical values of the considered entropy measures of the Kumaraswamy distribution at *b* = 2 and *δ* = 0.5.

*a*	*R*_*δ*_(*a*, *b*)	*HC*_*δ*_(*a*, *b*)	*A*_*δ*_(*a*, *b*)	*T*_*δ*_(*a*, *b*)	*AA*1_*δ*_(*a*, *b*)	*AA*2_*δ*_(*a*, *b*)
1.5	-0.026	-0.072	-0.059	-0.060	-0.125	0.374
2.0	-0.037	-0.100	-0.081	-0.083	-0.151	0.457
2.5	-0.059	-0.160	-0.128	-0.132	-0.180	0.555
3.0	-0.086	-0.229	-0.180	-0.189	-0.205	0.641
3.5	-0.115	-0.298	-0.232	-0.247	-0.225	0.713
4.0	-0.143	-0.366	-0.280	-0.303	-0.241	0.773
4.5	-0.170	-0.429	-0.324	-0.356	-0.255	0.824
5.0	-0.196	-0.489	-0.364	-0.405	-0.267	0.867
5.5	-0.222	-0.544	-0.400	-0.451	-0.276	0.904
6.0	-0.246	-0.595	-0.432	-0.493	-0.285	0.936

**Table 6:** Numerical values of the considered entropy measures of the Kumaraswamy distribution at *b* = 2 and *δ* = 1.5.

*a*	*R*_*δ*_(*a*, *b*)	*HC*_*δ*_(*a*, *b*)	*A*_*δ*_(*a*, *b*)	*T*_*δ*_(*a*, *b*)	*AA*1_*δ*_(*a*, *b*)	*AA*2_*δ*_(*a*, *b*)
1.5	-0.055	-0.221	-0.128	-0.130	-0.097	0.361
2.0	-0.075	-0.306	-0.177	-0.179	-0.113	0.416
2.5	-0.111	-0.466	-0.267	-0.273	-0.128	0.468
3.0	-0.151	-0.649	-0.369	-0.380	-0.140	0.508
3.5	-0.191	-0.838	-0.473	-0.491	-0.149	0.537
4.0	-0.228	-1.026	-0.574	-0.601	-0.156	0.561
4.5	-0.264	-1.211	-0.673	-0.709	-0.161	0.579
5.0	-0.297	-1.392	-0.768	-0.815	-0.166	0.594
5.5	-0.328	-1.568	-0.860	-0.918	-0.170	0.606
6.0	-0.358	-1.739	-0.948	-1.019	-0.173	0.617

**Table 7:** Numerical values of the considered entropy measures of the Kumaraswamy distribution at *b* = 2 and *δ* = 2.5.

*a*	*R*_*δ*_(*a*, *b*)	*HC*_*δ*_(*a*, *b*)	*A*_*δ*_(*a*, *b*)	*T*_*δ*_(*a*, *b*)	*AA*1_*δ*_(*a*, *b*)	*AA*2_*δ*_(*a*, *b*)
1.5	-0.070	-0.426	-0.170	-0.184	-0.081	0.378
2.0	-0.095	-0.600	-0.233	-0.259	-0.093	0.423
2.5	-0.137	-0.932	-0.346	-0.402	-0.103	0.462
3.0	-0.181	-1.340	-0.472	-0.578	-0.110	0.490
3.5	-0.223	-1.799	-0.602	-0.775	-0.116	0.511
4.0	-0.264	-2.297	-0.732	-0.990	-0.121	0.527
4.5	-0.301	-2.830	-0.860	-1.220	-0.124	0.539
5.0	-0.336	-3.392	-0.985	-1.462	-0.127	0.549
5.5	-0.369	-3.983	-1.108	-1.717	-0.129	0.557
6.0	-0.399	-4.600	-1.227	-1.982	-0.131	0.563

In view of Tables 2–7, the following comments can be formulated.

First, we recall that Tables 2–4 indicate the values of the entropy measures of the Kumaraswamy distribution for a fixed value of *a* and different values for *b* and *δ*. In this context,

- the Rényi, Havrda and Charvat, Arimoto, Tsallis and Awad and Alawneh1 entropy measures are decreasing when *b* is increasing while the Awad and Alawneh 2 entropy is increasing when *b* is increasing.
- the Rényi, Havrda and Charvat, Arimoto and Tsallis entropy measures are decreasing when *δ* is increasing while the Awad and Alawneh1 entropy is increasing when *δ* is increasing, but the Awad and Alawneh2 entropy is decreasing and increasing when *δ* is increasing.

Tables 5–7 show the values of the entropy of the Kumaraswamy distribution for a fixed value of *b* and different values for *a* and *δ*. In this setting,

- the Rényi, Havrda and Charvat, Arimoto, Tsallis and Awad and Alawneh1 entropy measures are decreasing when *a* is increasing while the Awad and Alawneh 2 entropy is increasing when *a* is increasing.
- the Rényi, Havrda and Charvat, Arimoto and Tsallis entropy measures are decreasing when *δ* is increasing while the Awad and Alawneh1 entropy is increasing when *δ* is increasing, but the Awad and Alawneh2 entropy is decreasing and increasing when *δ* is increasing.

## 3 Maximum likelihood estimation

The inference on the six considered entropy measures of the Kumaraswamy distribution is now investigated via the maximum likelihood technique. This technique is well-known and has proved itself in various modern studies such as those in [32–34].

### 3.1 Estimation of the entropy measures

The estimation of the parameters of the Kumaraswamy model through the maximum likelihood technique is well-known and the details can be found in [24]. The minimal theory is recalled below. Based on *n* values *x*_1_, …, *x*_*n*_ supposed to be observed from a random variable *X* with the Kumaraswamy distribution with parameters *a* and *b*, the maximum likelihood estimates (MLEs) of *a* and *b*, say $\hat{a}$ and $\hat{b}$, are defined by

$$\begin{array}{c} \left(\hat{a},\hat{b}\right)={\mathrm{argmax}}_{\left(a,b\right)\in {\left(0,+\infty \right)}^{2}}\ell \left(a,b\right), \end{array}$$

where *ℓ*(*a*, *b*) denotes the log-likelihood function specified by

$$\begin{array}{c} \ell \left(a,b\right)=n\log a+n\log b+\left(a-1\right)\sum_{i=1}^{n}\log x_{i}+\left(b-1\right)\sum_{i=1}^{n}\log(1-x_{i}^{a}). \end{array}$$

These MLEs are also the solutions of the two following equations according to *a* and *b*:

$$\begin{array}{c} \frac{\partial }{\partial a}\ell \left(a,b\right)=\frac{n}{a}+\sum_{i=1}^{n}\log x_{i}-\left(b-1\right)\sum_{i=1}^{n}\frac{x_{i}^{a}\log x_{i}}{1-x_{i}^{a}}=0,\;\frac{\partial }{\partial b}\ell \left(a,b\right)=\frac{n}{b}+\sum_{i=1}^{n}\log(1-x_{i}^{a})=0. \end{array}$$

That is, $\hat{a}$ and $\hat{b}$ satisfy the following simple relation:

$$\begin{array}{c} \hat{b}=-n{\left[\sum_{i=1}^{n}\log(1-x_{i}^{\hat{a}})\right]}^{-1}. \end{array}$$

Then, the properties of these MLEs follow from the usual maximum likelihood theory. In particular, thanks to the functional invariance of the MLEs, one can deduce easily the MLEs of the entropy measures. More concretely, based on the six entropy measures described in Subsection 2.2, $R_{\delta }(\hat{a},\hat{b})$ is the MLE of *R*_*δ*_(*a*, *b*), $HC_{\delta }(\hat{a},\hat{b})$ is the MLE of *HC*_*δ*_(*a*, *b*), $A_{\delta }(\hat{a},\hat{b})$ is the MLE of *A*_*δ*_(*a*, *b*), $T_{\delta }(\hat{a},\hat{b})$ is the MLE of *T*_*δ*_(*a*, *b*), $AA1_{\delta }(\hat{a},\hat{b})$ is the MLE of *AA*1_*δ*_(*a*, *b*), and $AA2_{\delta }(\hat{a},\hat{b})$ is the MLE of *AA*2_*δ*_(*a*, *b*).

### 3.2 Simulation

We now investigate the numerical behavior of the MLEs of the entropy measures via the use of simulated values. That is, we consider *N* = 5000 samples of values from a random variable *X* with the Kumaraswamy distribution of parameters *a* and *b* with different samples sizes; *n* = 100, 200, 300 and 1000 are considered. The following configurations on the parameters are considered: Configuration1: *a* = 3, *b* = 3 and *δ* ∈ *Ξ* with *Ξ* = {0.5, 1.5, 2.5}, and Configuration 2: *a* = 3, *b* = 5 and *δ* ∈ *Ξ*.

In each configuration, for each sample, the MLEs $\hat{a}$ and $\hat{b}$ are determined. Then, based on the *N* samples of fixed size, we determine the average of the *N* MLEs and use it to define the entropy estimates. The corresponding mean squared error (MSE) and mean deviation (MD) defined by the following generic formulas: MSE = sum(exact value—estimate)^2^ / N and MD = sum abs(exact value—estimate) / N, respectively, are also calculated. These assessment criteria are often used quite effectively to make a full comparison of models. In this regard, we can refer the reader to the useful works of [35–37].

The results on the Rényi entropy under Configurations 1 and 2 are given in Tables 8 and 9, respectively, results on the Havrda and Charvat entropy under Configurations 1 and 2 are indicated in Tables 10 and 11, respectively, results on the Arimoto entropy under Configurations 1 and 2 are presented in Tables 12 and 13, respectively, results on the Tsallis entropy under Configurations 1 and 2 are given in Tables 14 and 15, respectively, results on the Awad and Alawneh 1 entropy under Configurations 1 and 2 are given in Tables 16 and 17, respectively, and results on the Awad and Alawneh 2 entropy under Configurations 1 and 2 are indicated in Tables 18 and 19.

**Table 8:** Numerical values of the simulation related to the Rényi entropy for Configuration 1 (*a* = 3, *b* = 3).

*n*	*δ* = 0.5				*δ* = 1.5				*δ* = 2.5			
	*R*_*δ*_(*a*, *b*)	Estimate	MSE	MD	*R*_*δ*_(*a*, *b*)	Estimate	MSE	MD	*R*_*δ*_(*a*, *b*)	Estimate	MSE	MD
100	-0.2107	-0.2215	0.0020	0.0344	-0.3674	-0.3812	0.0039	0.0487	-0.4379	-0.4523	0.0046	0.0529
200		-0.2132	0.0009	0.0233		-0.3702	0.0018	0.0332		-0.4406	0.0021	0.0362
300		-0.2117	0.0005	0.0180		-0.3683	0.0010	0.0257		-0.4387	0.0012	0.0280
1000		-0.2113	0.0002	0.0103		-0.3680	0.0003	0.0147		-0.4385	0.0004	0.0160

**Table 9:** Numerical values of the simulation related to the Rényi entropy for Configuration 2 (*a* = 3, *b* = 5).

*n*	*δ* = 0.5				*δ* = 1.5				*δ* = 2.5			
	*R*_*δ*_(*a*, *b*)	Estimate	MSE	MD	*R*_*δ*_(*a*, *b*)	Estimate	MSE	MD	*R*_*δ*_(*a*, *b*)	Estimate	MSE	MD
100	-0.2753	-0.2800	0.0021	0.0371	-0.4504	-0.4553	0.0037	0.0488	-0.5258	-0.5307	0.0042	0.0522
200		-0.2781	0.0011	0.0260		-0.4535	0.0019	0.0343		-0.5289	0.0021	0.0366
300		-0.2802	0.0007	0.0218		-0.4564	0.0013	0.0287		-0.5321	0.0015	0.0306
1000		-0.2775	0.0002	0.0111		-0.4532	0.0003	0.0146		-0.5287	0.0004	0.0157

**Table 10:** Numerical values of the simulation related to the Havrda and Charvat entropy for Configuration 1 (*a* = 3, *b* = 3).

*n*	*δ* = 0.5				*δ* = 1.5				*δ* = 2.5			
	*HC*_*δ*_(*a*, *b*)	Estimate	MSE	MD	*HC*_*δ*_(*a*, *b*)	Estimate	MSE	MD	*HC*_*δ*_(*a*, *b*)	Estimate	MSE	MD
100	-0.2414	-0.2494	0.0021	0.0357	-0.6885	-0.7102	0.0154	0.0967	-1.4364	-1.4960	0.0913	0.2330
200		-0.2452	0.0013	0.0280		-0.6987	0.0094	0.0759		-1.4656	0.0550	0.1819
300		-0.2438	0.0007	0.0214		-0.6949	0.0054	0.0579		-1.4544	0.0311	0.1382
1000		-0.2433	0.0002	0.0113		-0.6937	0.0015	0.0307		-1.4496	0.0085	0.0731

**Table 11:** Numerical values of the simulation related to the Havrda and Charvat entropy for Configuration 2 (*a* = 3, *b* = 5).

*n*	*δ* = 0.5				*δ* = 1.5				*δ* = 2.5			
	*HC*_*δ*_(*a*, *b*)	Estimate	MSE	MD	*HC*_*δ*_(*a*, *b*)	Estimate	MSE	MD	*HC*_*δ*_(*a*, *b*)	Estimate	MSE	MD
100	-0.3104	-0.3188	0.0027	0.0400	-0.8623	-0.8864	0.0197	0.1085	-1.8572	-1.9299	0.1373	0.2816
200		-0.3134	0.0012	0.0282		-0.8708	0.0087	0.0761		-1.8836	0.0578	0.1953
300		-0.3151	0.0008	0.0227		-0.8753	0.0061	0.0613		-1.8939	0.0405	0.1576
1000		-0.3125	0.0003	0.0127		-0.8681	0.0019	0.0343		-1.8730	0.0123	0.0877

**Table 12:** Numerical values of the simulation related to the Arimoto entropy for Configuration 1 (*a* = 3, *b* = 3).

*n*	*δ* = 0.5				*δ* = 1.5				*δ* = 2.5			
	*A*_*δ*_(*a*, *b*)	Estimate	MSE	MD	*A*_*δ*_(*a*, *b*)	Estimate	MSE	MD	*A*_*δ*_(*a*, *b*)	Estimate	MSE	MD
100	-0.1900	-0.1980	0.0012	0.0275	-0.3908	-0.4073	0.0051	0.0553	-0.5008	-0.5216	0.0080	0.0695
200		-0.1943	0.0006	0.0195		-0.3997	0.0025	0.0390		-0.5120	0.0039	0.0489
300		-0.1925	0.0004	0.0160		-0.3959	0.0016	0.0321		-0.5071	0.0026	0.0403
1000		-0.1912	0.0001	0.0091		-0.3933	0.0005	0.0181		-0.5039	0.0008	0.0227

**Table 13:** Numerical values of the simulation related to the Arimoto entropy for Configuration 2 (*a* = 3, *b* = 5).

*n*	*δ* = 0.5				*δ* = 1.5				*δ* = 2.5			
	*A*_*δ*_(*a*, *b*)	Estimate	MSE	MD	*A*_*δ*_(*a*, *b*)	Estimate	MSE	MD	*A*_*δ*_(*a*, *b*)	Estimate	MSE	MD
100	-0.2406	-0.2476	0.0014	0.0297	-0.4859	-0.5013	0.0059	0.0607	-0.6182	-0.6380	0.0094	0.0767
200		-0.2426	0.0006	0.0197		-0.4904	0.0025	0.0401		-0.6240	0.0040	0.0507
300		-0.2420	0.0004	0.0161		-0.4891	0.0017	0.0327		-0.6222	0.0027	0.0413
1000		-0.2408	0.0001	0.0095		-0.4864	0.0006	0.0193		-0.6188	0.0009	0.0244

**Table 14:** Numerical values of the simulation related to the Tsallis entropy for Configuration 1 (*a* = 3, *b* = 3).

*n*	*δ* = 0.5				*δ* = 1.5				*δ* = 2.5			
	*T*_*δ*_(*a*, *b*)	Estimate	MSE	MD	*T*_*δ*_(*a*, *b*)	Estimate	MSE	MD	*T*_*δ*_(*a*, *b*)	Estimate	MSE	MD
100	-0.2000	-0.2083	0.0015	0.0304	-0.4033	-0.4193	0.0056	0.0584	-0.6190	-0.6508	0.0181	0.1038
200		-0.2041	0.0007	0.0210		-0.4111	0.0026	0.0402		-0.6344	0.0081	0.0710
300		-0.2043	0.0005	0.0180		-0.4116	0.0019	0.0345		-0.6348	0.0060	0.0608
1000		-0.2004	0.0001	0.0093		-0.4040	0.0005	0.0178		-0.6207	0.0015	0.0311

**Table 15:** Numerical values of the simulation related to the Tsallis entropy for Configuration 2 (*a* = 3, *b* = 5).

*n*	*δ* = 0.5				*δ* = 1.5				*δ* = 2.5			
	*T*_*δ*_(*a*, *b*)	Estimate	MSE	MD	*T*_*δ*_(*a*, *b*)	Estimate	MSE	MD	*T*_*δ*_(*a*, *b*)	Estimate	MSE	MD
100	-0.2572	-0.2633	0.0015	0.0308	-0.5051	-0.5173	0.0056	0.0589	-0.8004	-0.8271	0.0204	0.1120
200		-0.2604	0.0009	0.0232		-0.5117	0.0032	0.0443		-0.8150	0.0115	0.0838
300		-0.2590	0.0005	0.0183		-0.5088	0.0019	0.0348		-0.8086	0.0067	0.0656
1000		-0.2591	0.0002	0.0100		-0.5087	0.0006	0.0189		-0.8075	0.0020	0.0356

**Table 16:** Numerical values of the simulation related to the Awad and Alawneh 1 entropy for Configuration 1 (*a* = 3, *b* = 3).

*n*	*δ* = 0.5				*δ* = 1.5				*δ* = 2.5			
	*AA*1_*δ*_(*a*, *b*)	Estimate	MSE	MD	*AA*1_*δ*_(*a*, *b*)	Estimate	MSE	MD	*AA*1_*δ*_(*a*, *b*)	Estimate	MSE	MD
100	-0.48694	-0.48987	0.00102	0.02573	-0.33028	-0.33123	0.00021	0.01169	-0.25981	-0.26035	0.00009	0.00751
200		-0.48830	0.00057	0.01950		-0.33069	0.00012	0.00887		-0.26004	0.00005	0.00569
300		-0.48793	0.00034	0.01448		-0.33060	0.00007	0.00658		-0.26000	0.00003	0.00422
1000		-0.48703	0.00011	0.00825		-0.33028	0.00002	0.00375		-0.25980	0.00001	0.00241

**Table 17:** Numerical values of the simulation related to the Awad and Alawneh 1 entropy for Configuration 2 (*a* = 3, *b* = 5).

*n*	*δ* = 0.5				*δ* = 1.5				*δ* = 2.5			
	*AA*1_*δ*_(*a*, *b*)	Estimate	MSE	MD	*AA*1_*δ*_(*a*, *b*)	Estimate	MSE	MD	*AA*1_*δ*_(*a*, *b*)	Estimate	MSE	MD
100	-0.51891	-0.52148	0.00074	0.02153	-0.34379	-0.34465	0.00014	0.00942	-0.26837	-0.26886	0.00006	0.00598
200		-0.52091	0.00038	0.01553		-0.34456	0.00007	0.00677		-0.26883	0.00003	0.00430
300		-0.52043	0.00023	0.01221		-0.34438	0.00004	0.00534		-0.26873	0.00002	0.00339
1000		-0.51951	0.00007	0.00660		-0.34403	0.00001	0.00288		-0.26852	0.00001	0.00183

**Table 18:** Numerical values of the simulation related to the Awad and Alawneh 2 entropy for Configuration 1 (*a* = 3, *b* = 3).

*n*	*δ* = 0.5				*δ* = 1.5				*δ* = 2.5			
	*AA*2_*δ*_(*a*, *b*)	Estimate	MSE	MD	*AA*2_*δ*_(*a*, *b*)	Estimate	MSE	MD	*AA*2_*δ*_(*a*, *b*)	Estimate	MSE	MD
100	0.66553	0.66962	0.00226	0.03816	0.51972	0.52074	0.00041	0.01628	0.49927	0.49983	0.00020	0.01135
200		0.66502	0.00127	0.02792		0.51909	0.00023	0.01197		0.49874	0.00011	0.00835
300		0.66668	0.00084	0.02284		0.51994	0.00015	0.00977		0.49937	0.00008	0.00681
1000		0.66621	0.00027	0.01301		0.51993	0.00005	0.00556		0.49940	0.00002	0.00387

**Table 19:** Numerical values of the simulation related to the Awad and Alawneh 2 entropy for Configuration 2 (*a* = 3, *b* = 5).

*n*	*δ* = 0.5				*δ* = 1.5				*δ* = 2.5			
	*AA*2_*δ*_(*a*, *b*)	Estimate	MSE	MD	*AA*2_*δ*_(*a*, *b*)	Estimate	MSE	MD	*AA*2_*δ*_(*a*, *b*)	Estimate	MSE	MD
100	0.71515	0.72043	0.00166	0.03256	0.53922	0.54086	0.00026	0.01303	0.51263	0.51366	0.00012	0.00892
200		0.71595	0.00080	0.02300		0.53930	0.00013	0.00925		0.51264	0.00006	0.00634
300		0.71711	0.00053	0.01825		0.53985	0.00008	0.00732		0.51303	0.00004	0.00502
1000		0.71663	0.00017	0.01059		0.53976	0.00003	0.00424		0.51299	0.00001	0.00290

Based on Tables 8–19, in all the situations, we see that the MLEs of the entropy measures are close to the target values and, as anticipated, the MSEs and MDs decrease and approach 0 as *n* increases. This proves the accuracy of the proposed estimation methods in the context of the Kumaraswamy distribution. Also, one can notice that the MSEs and MDs increase as *δ* increases.

For a visual approach, the behavior of the MSEs and MDs are illustrated in Figs 1–12, for the Rényi, Havrda and Charvat, Arimoto, Tsallis, Awad and Alawneh 1 and Awad and Alawneh 2 entropy measures following the settings of Tables 8–19, respectively.

**Fig 1:**
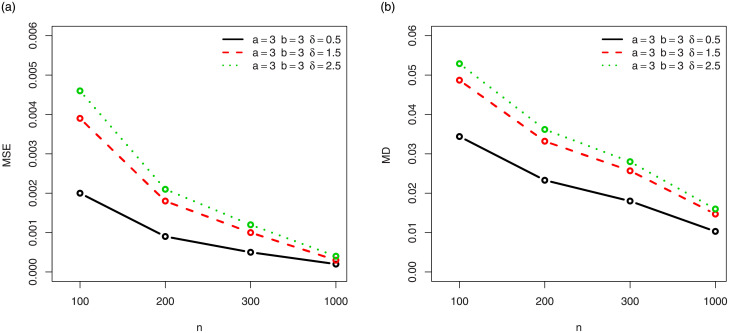
Plots of the (a) MSEs and (b) MDs for the Rényi entropy in the setting of Table 8.

**Fig 2:**
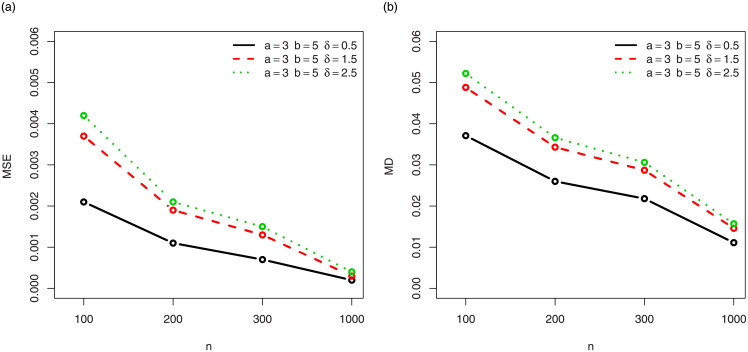
Plots of the (a) MSEs and (b) MDs for the Rényi entropy in the setting of Table 9.

**Fig 3:**
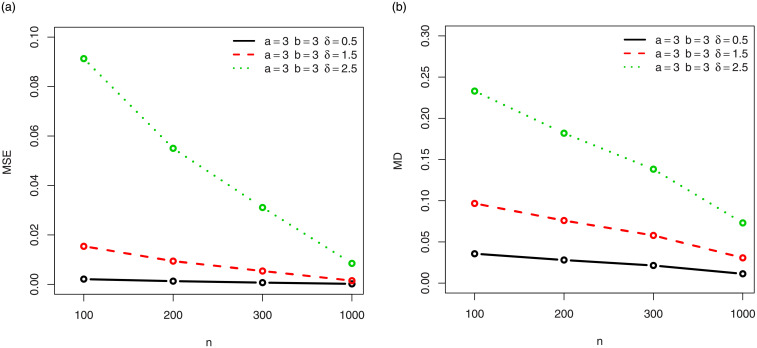
Plots of the (a) MSEs and (b) MDs for the Havrda and Chardat entropy in the setting of Table 10.

**Fig 4:**
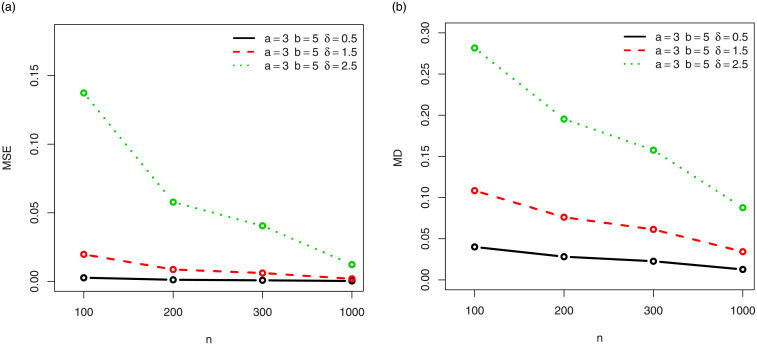
Plots of the (a) MSEs and (b) MDs for the Havrda and Chardat entropy in the setting of Table 11.

**Fig 5:**
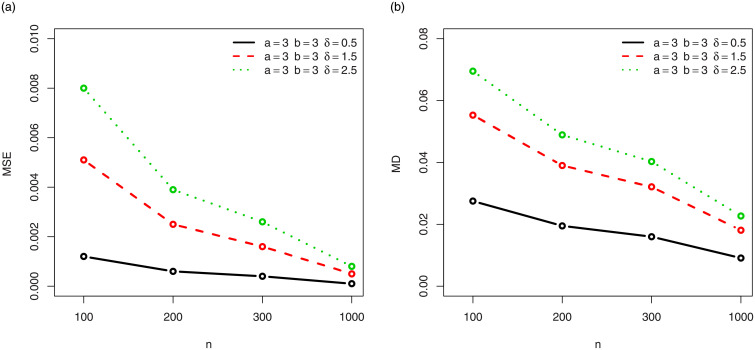
Plots of the (a) MSEs and (b) MDs for the Arimoto entropy in the setting of Table 12.

**Fig 6:**
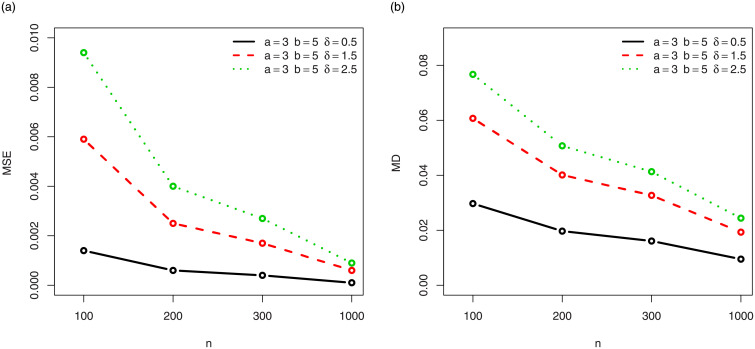
Plots of the (a) MSEs and (b) MDs for the Arimoto entropy in the setting of Table 13.

**Fig 7:**
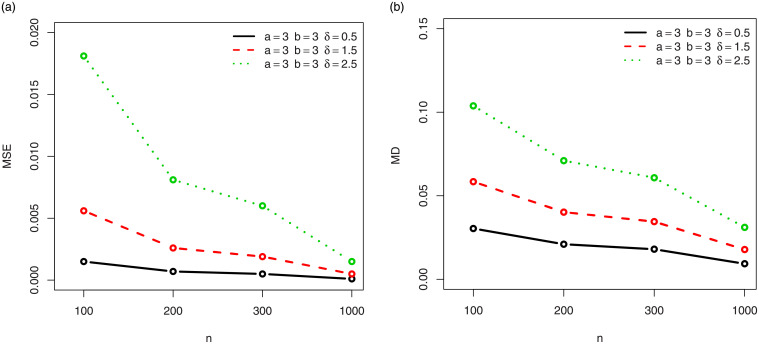
Plots of the (a) MSEs and (b) MDs for the Tsallis entropy in the setting of Table 14.

**Fig 8:**
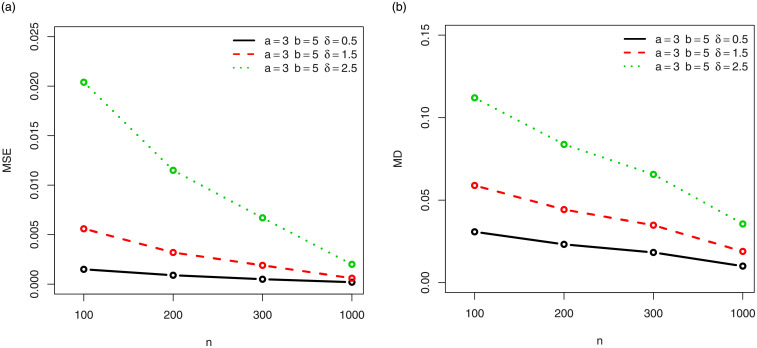
Plots of the (a) MSEs and (b) MDs for the Tsallis entropy in the setting of Table 15.

**Fig 9:**
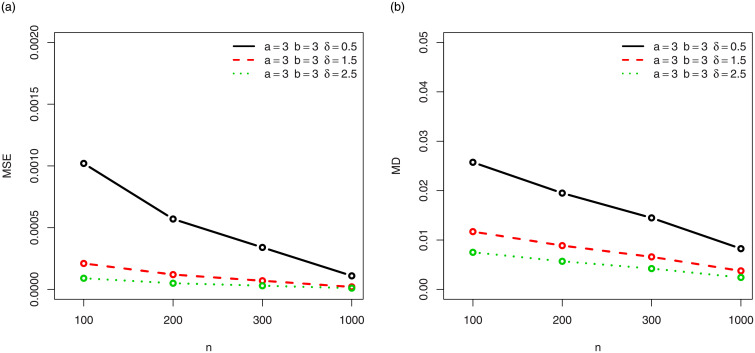
Plots of the (a) MSEs and (b) MDs for the Awad and Alawneh 1 entropy in the setting of Table 16.

**Fig 10:**
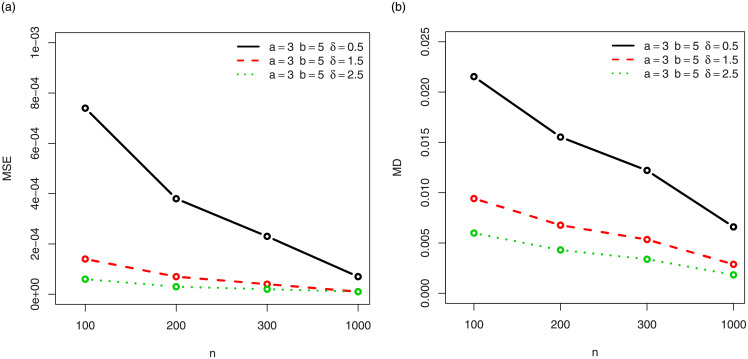
Plots of the (a) MSEs and (b) MDs for the Awad and Alawneh 1 entropy in the setting of Table 17.

**Fig 11:**
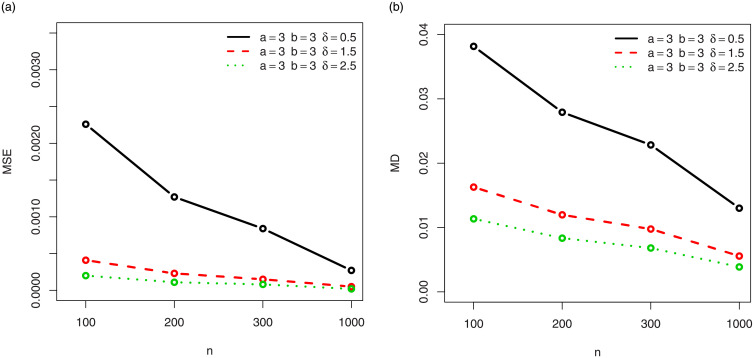
Plots of the (a) MSEs and (b) MDs for the Awad and Alawneh 2 entropy in the setting of Table 18.

**Fig 12:**
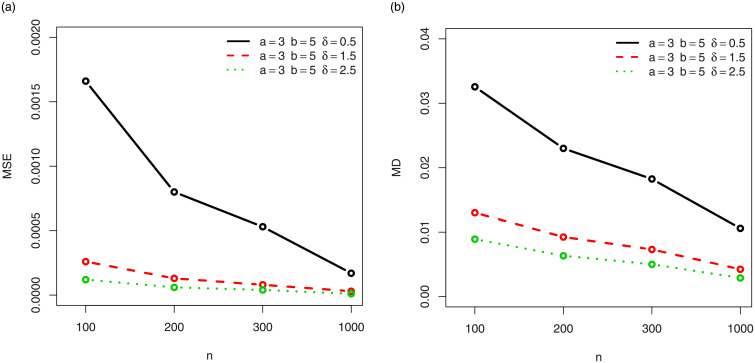
Plots of the (a) MSEs and (b) MDs for the Awad and Alawneh 2 entropy in the setting of Table 19.

Figs 1–12 support the claims formulated about the results of Tables 8–19.

### 3.3 Illustrative examples

In this Section, two real life data sets are used to illustrate the proposed methodology. The considered data sets are described below.

#### The first data set

The data set consists of 48 rock samples from an oil reservoir. It corresponds to twelve oil tank cores that were sampled by four cross sections. Each core was measured for permeability and each cross section has the following variables: total pore area, total pore perimeter, and shape. We analyze the perimeter of the shape by a squared variable (area). It has been analyzed by [38], among others. Explicitely, the data set is: {0.0903296, 0.2036540, 0.2043140, 0.2808870, 0.1976530, 0.3286410, 0.1486220, 0.1623940, 0.2627270, 0.1794550, 0.3266350, 0.2300810, 0.1833120, 0.1509440, 0.2000710, 0.1918020, 0.1541920, 0.4641250, 0.1170630, 0.1481410, 0.1448100, 0.1330830, 0.2760160, 0.4204770, 0.1224170, 0.2285950, 0.1138520, 0.2252140, 0.1769690, 0.2007440, 0.1670450, 0.2316230, 0.2910290, 0.3412730, 0.4387120, 0.2626510, 0.1896510, 0.1725670, 0.2400770, 0.3116460, 0.1635860, 0.1824530, 0.1641270, 0.1534810, 0.1618650, 0.2760160, 0.2538320, 0.2004470}.

#### The second data set

This data set contains 20 observations of flood data. It was analyzed by [39]. The data set is listed as follows: {0.265, 0.392, 0.297, 0.3235, 0.402, 0.269, 0.315, 0.654, 0.338, 0.379, 0.418, 0.423, 0.379, 0.412, 0.416, 0.449, 0.484, 0.494, 0.613, 0.74}.

In order to check the adequateness of the Kumaraswamy distribution to these data, we apply the Kolmogorov-Smirnov test. We find p-value 0.2092 and p-value = 0.3359 for the first and second data sets, respectively. Since both satisfy p-values >0.05, the two considered data set are in adequateness with the Kumaraswamy distribution.

Now, Tables 20 and 21 present the estimations of the six entropy measures considered in Subsection 2.2, following the methodology described in Subsection 3.1, for the first and second data sets, respectively.

**Table 20:** Estimates of the considered entropy measures with different values of *δ* for the first data set.

*δ*	*R*_*δ*_(*a*, *b*)	*HC*_*δ*_	*A*_*δ*_(*a*, *b*)	*T*_*δ*_(*a*, *b*)	*AA*1_*δ*_(*a*, *b*)	*AA*2_*δ*_(*a*, *b*)
0.5	-0.395	-0.881	-0.597	-0.730	-2.668	0.807
1.5	-0.487	-2.570	-1.361	-1.505	1.668	0.567
2.5	-0.523	-7.887	-1.768	-3.399	0.438	0.530

**Table 21:** Estimates of the considered entropy measures with different values of *δ* for the second data set.

*δ*	*R*_*δ*_(*a*, *b*)	*HC*_*δ*_	*A*_*δ*_(*a*, *b*)	*T*_*δ*_(*a*, *b*)	*AA*1_*δ*_(*a*, *b*)	*AA*2_*δ*_(*a*, *b*)
0.5	-0.201	-0.499	-0.370	-0.413	-2.663	0.801
1.5	-0.291	-1.359	-0.751	-0.796	1.666	0.570
2.5	-0.327	-3.245	-0.953	-1.399	0.437	0.533

We can notice that, under our framework, the Rényi, Havrda and Charvat, Arimoto, Tsallis, Awad and Alawneh 2 entropy measures are decreasing when *δ* is increasing while the Awad and Alawneh 1 entropy is increasing when *δ* is increasing.

Tour knowledge, it is the first time that the entropy of the uncertainty behind these data sets are evaluated. They can be taken into account for further statistical analysis in the future.

## 4 Conclusion

For the first time, this article proposed a special focus on the entropy of the Kumaraswamy distribution. Both theoretical and practical aspects were covered, though complementary works. In particular, six different entropy measures were investigated. After determining the closed-form expressions of these measures, an estimation strategy was developed to evaluate them in a practical setting. A simulation study ensured the convergence of the obtained estimates. Two real-life data sets are used to show how the related entropy can be concretely estimated. The finding of this study aims to be applied by the statistician to assess the entropy of diverse data with values on the unit interval, such as modern rate, percentage and proportion type data.

The limitation of current research remains on the classicity of the statistical framework considered. Directions for future research include the estimation of the entropy of the Kumaraswamy distribution in more sophisticated statistical schemes with physical motivations, such as the progressive type II censoring scheme, generalized progressively hybrid censoring scheme, etc., or taking into account generalized versions of the Kumaraswamy distribution, such as the one proposed by [40].
